# Short-term culture of ovarian cortex pieces to assess the cryopreservation outcome in wild felids for genome conservation

**DOI:** 10.1186/1746-6148-9-37

**Published:** 2013-02-22

**Authors:** Caterina Wiedemann, Jennifer Zahmel, Katarina Jewgenow

**Affiliations:** 1 Leibniz Institute for Zoo and Wildlife Research (IZW), PF 700430, Berlin, 10324, Germany

**Keywords:** Wild felids, Domestic cat, Cryopreservation, Ovarian cortex, Ovarian tissue culture, Primordial follicles

## Abstract

**Background:**

Cryopreservation of ovarian tissue has the potential to preserve female germ cells of endangered mammals. In the present study, a freezing protocol successfully used for human tissue, was adapted for preserving ovarian tissue of domestic and non-domestic felids. Ovaries from non-domestic felid species were obtained from seven freshly euthanized and two recently deceased wild felids kept in different European Zoos. In addition, ovaries from domestic cats were obtained after ovariectomy from local veterinary clinics for methological adaptations.

Ovarian cortex was dissected and uniform sized pieces of 2 mm diameter were obtained. Using a slow freezing protocol (-0.3°C per min) in 1.5 mol/L ethylene glycol, 0.1 mol/L sucrose, the pieces were cultured for up to 14 days both before and after cryopreservation. The integrity of primordial follicles was assessed by histology, and the impact of different protein sources (FCS or BSA) and Vitamin C was determined during two weeks of culture.

**Results and conclusion:**

During culture the number of primordial follicles decreased within the ovarian pieces (p < 0.05). This effect was less pronounced when FCS was used as the protein source instead of BSA. Supplementation with Vitamin C had a detrimental effect on follicle survival. Since the procedure of cryopreservation had no effect on the follicle survival after one week of culture we conclude that the freezing protocol was suitable for felids. This is the first report of preserving a huge amount of follicles within ovarian tissue by slow freezing performed in several wild feline species.

## Background

With the exception of the domestic cat all other felid species are listed on the Red list of IUCN for endangered species [1]. In the wild, population decline of felids is mainly caused by human impacts (poaching, habitat destruction) and/or disease transmission, e.g. Feline immunodeficiency virus or tuberculosis. The situation of felids in zoos is comparably alarming. The number of potential breeders within captive populations is often compromised by husbandry issues such as the size of enclosures, incompatible pairing and inbreeding. In addition, the restocking of zoo populations with newly captured wild animals is not possible because of import/export restrictions by the Convention of International Trade in Endangered Species of Wild Fauna and Flora (CITES, [2]).

Assisted reproductive technology (ART) has been suggested as a significant tool in overcoming breeding problems and broadening the gene pool for species conservation [3]. The modern methodology of ART includes hormonal regulation of cycling activity, artificial insemination, aspiration of oocytes, collection of spermatozoa, in vitro production of embryos, embryo transfer and, finally, genetic resource banking. Genetic resource banking is defined as the storage of gametes (sperm and oocytes) and embryos from threatened populations with the deliberate intention to use them in breeding programs at the time of ones choosing [4]. In combination with artificial insemination, in vitro fertilization and embryo transfer, it allows breeding of behaviorally or medically incompatible pairs independently of time and location. Although genetic resource banking has been propagated for safeguarding threatened wildlife species for many years [4,5], little progress has been made to incorporate these technologies into zoo breeding programs. Often the zoos are not aware of the fact that ovaries of every animal contain viable oocytes that can be preserved providing appropriate collection, handling and freezing methods [6]. In many species, oocytes can tolerate cryopreservation very well irrespective of whether they are frozen within a whole ovary, as ovarian pieces, or as individual, isolated follicles [7]. The availability of mature oocytes is the limiting factor of ART. The main follicle pool of a cat ovary consists of 90 to 99% primordial follicles (PF) located within the cortex [8]. To utilize the oocyte reserve from female cat gonads, slow freezing of ovarian cortex, similar to that already successfully used for human tissue [9], was tried. In human, methods and media for cryopreservation is investigated and published very intensively. The most important issue of ovarian tissue freezing is the application for fertility restoration after chemo- and radiotherapy in tumor patients. Since 2004, more than 25 human babies have been born [10]. Live birth after ovarian tissue cryopreservation and re-transplantation was also reported for mice [11], goats [12], and ewes [13]. Thus, cryopreservation of ovarian cortex might be considered for genome conservation and oocyte retrieval in felids. Bosch et al. [14] demonstrated the successful cryopreservation of domestic cat ovarian cortex in conjunction with xenotransplantation into nude mice. However, feline oocytes seem to express a higher sensitivity towards cryopreservation protocols [15,16] than other mammalian oocytes.

The aim of the present study was to adapt a cryopreservation protocol, successfully applied in human reproductive medicine [17], to preserve female germ cells from feline species. Ovaries from non-domestic cats were obtained from several European Zoos participating in the Felid Gametes Rescue Project [6]. The domestic cat was used for methodological adaptation because it is a proven model species for the improvement of ART for all felids and ovaries can be obtained on a regular basis in contrast to wild species.

## Methods

All chemicals were obtained from Sigma-Aldrich (Taufkirchen, Germany) unless otherwise stated.

Ovaries from non-domestic felid species were obtained from seven freshly euthanized and two recently deceased wild felids kept in different European Zoos (Table 1). Animals were euthanized for medical or management reasons, each individual zoo was responsible for the approval. In none of the cases the animals were euthanized for purpose of this study. Immediately after death the ovaries were placed into PBS (phosphate buffered saline) and shipped to Berlin via an overnight express service. The ovaries were processed within 6 to 48 h post mortem (Table 1). Ovaries from domestic cats (*Felis silvestris catus,* n = 7) were obtained after ovariectomy from local veterinary clinics. Ovaries were placed into 50 mL Greiner tubes (Greiner bio-one, Frickenhausen, Germany) containing Minimum Essential Medium Eagle HEPES Modification supplemented with 3 mg BSA (bovine serum albumin) mL^-1^ (Merck, Darmstadt, Germany) and 1× Antibiotic Antimycotic Solution and transferred to the laboratory at 4°C within 2 to 4 h. After arrival the ovaries were processed immediately.

**Table 1:** Origin, age, causes of death of wild felid species

**Species**		**Origin**	**Age [years]**	**Ovary sampling after**	**Transportation time [hours]**
African lion	(*Panthera leo*)	Givskud Zoo	7	euthanasia	10
Amur leopard	(*Panthera pardus orientalis*)	Copenhagen Zoo	1	euthanasia	10
Black-footed cat	(*Felis nigripes*)	Wuppertal Zoo	1	euthanasia	16
Geoffroy's cat	(*Leopardus geoffroyi*)	Tierpark Berlin	12	euthanasia	24
Northern Chinese leopard	(*Panthera pardus japonensis*)	Ebeltoft Ree Park	3	euthanasia	12
Oncilla	(*Leopardus tigrinus*)	Dortmund Zoo	14	euthanasia	22
Rusty-spotted cat	(*Prionailurus rubiginosus*)	Frankfurt Zoo	5	post mortem	48
Serval (2)	(*Leptailurus serval*)	Tierpark Berlin	–	post mortem (stillbirth)	24
Sumatran tiger	(*Panthera tigris sumatrae*)	Tierpark Berlin	13	euthanasia	24

### Ovarian cortex preparation

The ovaries were washed and cut into two halves in 60 × 15 petri dishes (1 well, Thermo Fischer Scientific, Nunc IVF petri dish, non-treated surface, Braunschweig, Germany) containing wash media consisted of Mc Coys 5a medium modified (M8403) supplemented with 20 mM HEPES, 3 mM glutamine, 0.1 mg/mL gentamicin and BSA or FCS (fetal calve serum). The protein content was varied within the experiments.

By using a scalpel (scalpel handle no. 3, blade no. 10), the medulla was removed until a thin layer of ovarian cortex (approximately 200 μm thick) was obtained. The nearly transparent ovarian cortex was placed into fresh wash media and punched with 2 mm diameter Biopsy punches (pfm, Cologne, Germany) to obtain similar size pieces. The pieces were allocated randomly into two groups. Half of the pieces were used for culture and were further processed at room temperature. The pieces of the other group were chosen for cryopreservation and placed on ice water (0°C).

### In vitro culture

For in vitro culture, at most six ovarian pieces were placed into Easyflasks (Thermo Fisher Scientific, Nunclon™ Surface, 25 cm^2^, Braunschweig, Germany) with 7 mL culture medium according to Telfer et al. [17] and cultured on a shaker in a CO_2_-incubator at 5% CO_2_ and 5% O_2_ at 38.5°C. No media change was performed. At the sampling days, all pieces of one Easyflask were removed and fixed for follicle integrity assessment.

### Cryopreservation

Cryopreservation was performed by slow freezing according to Kvist et al. [16] in cryomedium consisting of PBS supplemented with 1.5 mol/L ethylene glycol (Roth, Karlsruhe, Germany), 0.1 mol/L sucrose and BSA or FCS (see experimental design 2.). Two different programmable cryo devices were used. The Kryo 10-16/III (MG, Krefeld, Germany) was equipped with 2 mL cryovials (Greiner bio-one, Frickenhausen, Germany) containing at most six ovarian pieces in 1 mL cryomedium, whereas the Labotect Cryo Unit (LCU, Göttingen, Germany) was applied to freeze samples in straws containing 3 to 4 pieces. The commercial available 250 μL straws (Minitüb, Tiefenbach, Germany) were shortened to a length of 6 cm and the plug was gently moved into the centre. After pre-filling with cryomedium (150 μL) the straws were sealed on one side and kept at 4°C. After placement of 3 to 4 ovarian pieces on top of the plug the upper site was sealed as well. With exception of ovaries from the Sumatran tiger, all other feline samples were frozen by using the LCU.

Ovarian samples were equilibrated in 50 mL cryomedium in Greiner tubes, on a shaker for 15 min on ice water before transferring them into freezing vials and placing them into the cryo device. The following cryoprotocol was used: start at 1°C, hold for 10 min, cooling -2°C per min to -4°C, cooling -0.2°C per min to -8°C, 10 min holding time for seeding, cooling with -0.3°C per min to -40°C and finally -10°C/min to -140°C followed by plunging samples into liquid nitrogen. The Kryo 10-16/III freezer was not able to reach temperature of -140° C. Therefore the samples were plunged in liquid nitrogen when the temperature reached a plateau (at about -40 to -50°C). Seeding of cryo vials (Kryo 10-16/III freezer) was performed manually at -8°C by touching the vials with an ice-cold forceps. The ice crystal formation in the straws happened spontaneously in LCU because of the vertical position of the straws above the liquid nitrogen vapor.

### Thawing

The samples were thawed rapidly by shaking them in a water bath at 37°C for 10 s for straws and 1.5 min for cryo vials, respectively. The cryomedium was removed by a three-step diluting procedure: 5 min in PBS with 0.75 mol/L ethylene glycol and 0.1 mol/L sucrose, 5 min in PBS with 0.1 mol/L sucrose and 5 min in PBS. Before starting the culture, the thawed pieces were washed twice in wash media.

### Histology of ovarian cortex

Before and after freezing and at different time periods of culture (2 days, 7 days, 14 days) ovarian pieces were fixed in Bouin solution (Sigma HT10132) for 24 h, afterwards moved to PBS and embedded in paraffin by a standard histological procedure. The ovarian pieces were placed vertically in paraffin wax and serial cuttings of 3 μm thickness were performed. Every tenth section was mounted on glass slides (Engelbrecht, Edermünde, Germany) and stained with haematoxylin and eosin (Merck, Darmstadt, Germany).

### Follicle measurements

**Figure 1:**
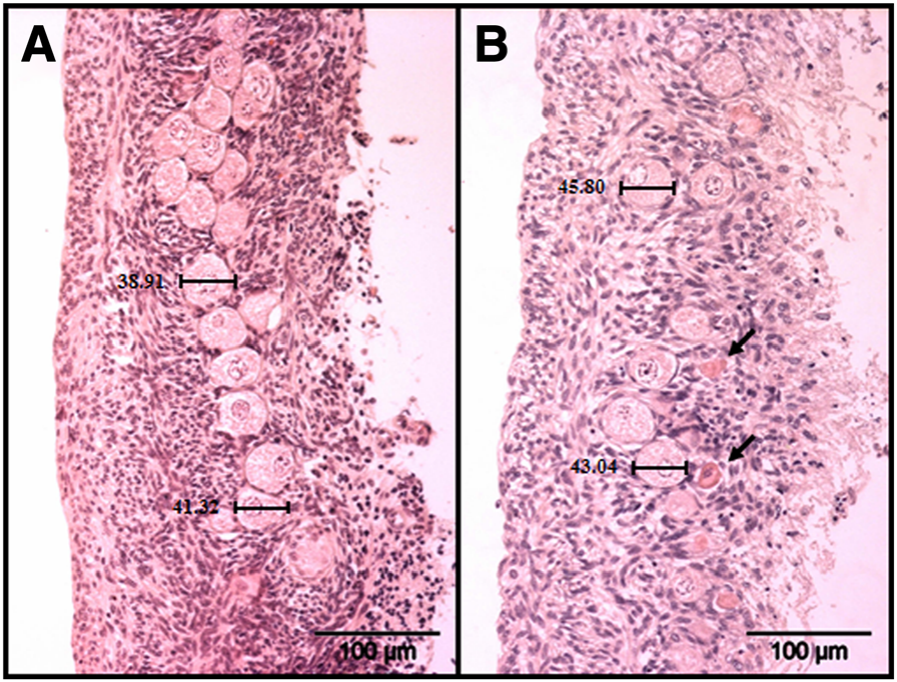
**Histological sections of ovarian cortex from the domestic cat before A) day 0 and after culture B) day 7.** Follicle diameter was determined by measurement of maximum diameter with computer assisted image analysis Cell^^^D. Arrows indicate shrunken follicles after culture.

For each ovarian cortex piece of the domestic cat pool and the Amur leopard all follicles were measured under a microscope (Axiovert, Carl Zeiss, Jena, Germany) by using the computer software Cell^^^D (Olympus, analySIS, Hamburg, Germany). The follicular diameter was determined measuring the maximum dimension within the follicular basal membrane (Figure 1). Follicles were divided in consideration of follicle morphology in different size groups: < 30 μm truncated or shrunken follicles, 30 to 40 μm PF (flat granulosa cells), 40 to 50 μm primary follicles (cubic granulosa cells) and > 50 μm secondary follicles (more than 1 layer of cubic granulosa cells). The results are presented by relative number of different size class follicles determined in at least two pieces. As an alternative approach, only 40 follicles were measured and the number of sections needed for 30 primordial follicles was noted. Then, a calculated number of follicles per piece (CFN) were determined by extrapolation of the number of sections (nSections) needed for 30 primordial follicles (nPF) to the whole dimension of the round ovarian piece (2 mm = Diameter_ovarian_piece_):

$$\begin{array}{l} \mathrm{CFN}=\frac{\pi }{4}\frac{\mathrm{Diamete}r_{\mathrm{ovarian}\_\mathrm{piece}}*\mathrm{nPF}}{\mathrm{nSections}*\mathrm{Sectio}n_{\mathrm{dis}\tan\mathrm{ce}}}\\ \mathrm{CFN}=\frac{\pi }{4}\frac{2000\mathrm{\mu m}*30}{\mathrm{nSections}*30\mathrm{\mu m}} \end{array}$$

For all examined felid species, the size of 40 separate follicles in at least two pieces was determined together with the mean number of sections needed for reaching the necessary follicles number. The distance between measured follicles represented 30 μm (Section_distance_). The factor π/4 is required for correction from a quadratic to a circular area. A correction of histological counting according to Abercrombie [18] was neglected, because all tissue pieces were treated identically.

### Experimental design

1. *Analysis of follicular distribution during two weeks culture period in the domestic cat*

To assess the impact of in vitro culture on the distribution of follicles within the ovarian piece we pooled samples from seven domestic cats and cultured them for 14 days. Pieces were removed from culture on day 2, 7 and day 14. The overall number of follicles per piece and the size distribution was determined before culture and for each sampling day. In addition, the number of histological section which was necessary to measure a total of 30 primordial follicles was determined to estimate the CFN (calculated follicle number per piece). In contrast to the overall number of follicles, the percentage of PF in only 40 follicles was determined separately.

2. *Influence of media composition on primordial follicle survival*

Ovarian pieces from an Amur leopard were used to analyze the impact of protein source on follicles survival during prolonged culture. For this respect, the ovarian pieces were supplemented with either 0.1% BSA or 10% FCS, respectively. The mean follicle number per piece, the follicles size distribution as well as the CFN (based on 30 primordial follicles) were determined at day 0, day 2, day 7 and day 14 of culture. In addition to experiment 1, samples of the pool from 7 domestic cats were cultured in Mc Coys 5a medium supplemented with Vitamin C. Before and after 2, 7 and 14 days of culture the percentage of follicles was determined as described before.

3. *Standard sampling protocol for all other felid species*

The standard ovarian culture and freezing protocol was established as a result of experiment 1 and 2. Ovarian pieces were subjected to culture medium supplemented with 20% FCS and without Vitamin C. The histology was performed on samples obtained before and after freezing on day 0 and day 7 of culture. Because no histological differences were found between day 0 before and after cryopreservation we treated day 0 as one group. For each sampling day, the calculated number of follicles was estimated based on 30 primordial follicles, which were measured for size.

### Statistical analysis

Statistical analysis was performed by using the statistical program InStat3 (GraphPad Software, Inc., California, USA). The results of total follicle distribution during culture and the relative primordial follicle survival in different culture experiments in ovarian cortex pieces were analyzed by one-way ANOVA. If they passed the normality test, the Tukey-Kramer Multiple Comparisons test was used. In case of Amur leopard experiment the normality test was not passed, therefore we performed the non-parametric Dunn’s Multiple Comparisons test. Chi-square test was applied for comparisons of primordial survival rates. Thus, the impact of cryopreservation was analyzed by comparing survival rates on day 0 and 7 after culture and on day 7 before and after freezing. For all statistical analysis, differences were regarded to be significant if p < 0.05.

## Results

### Analysis of follicular distribution during the two week culture period

During the two week culture, there was a change in both the total number of follicles and the percentage of follicles in each size group within the ovarian pieces. Table 2 shows the mean number (± sd) of all follicles and the percentage of follicles within different follicle groups of pooled domestic cat samples. The mean follicle number per piece (654 ± 427 follicles) decreased by 36% after two days. The prolonged culture was accompanied by further loss to 256 ± 322 and 164 ± 136 follicles after seven and 14 days, respectively. The calculated follicle number per piece (CFN) decreased over time, and the distribution of size groups shifted markedly.

**Table 2:** Follicle distribution in pooled domestic cat ovarian cortex pieces over 14 days in vitro culture without supplementation (control)

**A)**	**Mean number (%)**			
**Follicle group**	**Day 0 (n = 6)**	**Day 2 (n = 6)**	**Day 7 (n = 6)**	**Day 14 (n = 3)**
all follicles	654 ± 427	419 ± 240	256 ± 322	164 ± 136
shrunken/truncated follicles	142 ± 95 (21,81%)	142 ± 97 (36,62%)	92 ± 88 (40,70%)	126 ± 110 (66,72%)
primordial follicles (%PF)	378 ± 268 (56,20%)^a^	185 ± 137 (41,45%)^a,b^	111 ± 153 (39,68%)^a,b^	26 ± 20 (24,46%)^b^
primary follicles	125 ± 63 (20,88%)^a^	71 ± 60 (16,50%)^a,b^	46 ± 88 (14,92%)^a,b^	9 ± 8 (8,23%)^b^
secondary follicles	8 ± 7 (1,11%)^a,b^	21 ± 11 (5,43%)^a^	7 ± 7 (4,69%)^a,b^	2 ± 3^b^ (0,59%)^b^
%PF_40_ (measured follicles)	59,58% (240)	45,83% (240)	39,17% (210)	17,50% (88)
CFN	496	242	116	121

a, b - different subscript indicate for significant differences.

Before culture (day 0), the follicle population consisted mainly of PF (30 to 40 μm), which decreased during culture to mean values of 26 ± 20 in each ovarian cortex piece (p < 0.05). In parallel, the percentage of shrunken follicles (< 30 μm) increased during the culture to 67% of all measured follicles. Additionally, 21% of primary follicles were found in the ovarian cortex pieces at day 0, and this number decreased during the culture to values of 8% at the end of the study (p < 0.05). Only a few secondary follicles were found in the ovarian cortex pieces.

The percentage of primordial follicles (%PF) was determined according to the total number of follicles (Table 2). In addition, 88–240 follicles were measured (40 follicles per piece, n = 3–6 pieces) to determine the proportion of PF, when only 40 follicles per piece were considered (%PF_40_). As seen in Table 2, there was no difference between %PF and %PF_40_ with 60% and 56% at day 0, 46% and 42% at day 2, 39% and 40% at day 7, as well as 18% and 25% at day 14 for all follicles and 40 measured follicles, respectively.

### Influence of media composition on primordial follicle survival

#### BSA vs. FCS

Table 3 presents a 14 day culture experiment performed on ovarian cortex pieces from an Amur leopard with culture media containing different protein sources (BSA vs. FCS). All parameters that were determined for FCS supplementation (Table 3A) stayed unchanged during culture (no statistical differences). Only in secondary follicles a statistical decrease were found after 14 days of culture (p < 0.05). In contrast, BSA supplementation (Table 3B) caused a significant decrease in mean follicles number per piece, %PF (based on all follicles) and percentage of secondary follicles between day 0 and day 14. The percentage of PF determined in 40 follicles per piece (%PF_40_), however, did not reflect the follicular loss because of the high variation and very low number of follicles left after 14 days. At day 14, the overall follicle number in two ovarian cortex pieces was only 24 ± 28, but still 10 viable PF were found in the BSA group at this sampling day. As also shown for the domestic cat (Table 2) the percentage of PF for the Amur leopard were identical using different determination methods (%PF or %PF_40_).

**Table 3:** Follicle distribution in ovarian cortex pieces from an Amur leopard of 14 days in vitro culture with A) FCS and B) BSA supplementation

**A)**	**Mean number (%)**			
**Follicle group**	**Day 0**	**Day 2**	**Day 7**	**Day 14**
	**(n = 2)**	**(n = 3)**	**(n = 3)**	**(n = 3)**
all follicles	313 ± 123	98 ± 69	62 ± 16	55 ± 4
shrunken/truncated follicles	29 ± 9 (9,24%)	20 ± 11 (25,97%)	18 ± 7 (28,45%)	47 ± 6 (85,41%)
primordial follicles (%PF)	129 ± 57 (40,82%)	43 ± 33 (38,54%)	29 ± 8 (46,27%)	6 ± 4 (11,43%)
primary follicles	107 ± 37 (34,32%)	21 ± 19 (16,77%)	9 ± 3 (15,08%)	1 ± 1 (1,89%)
secondary follicles	49 ± 20 (15,62%)^a^	14 ± 8 (18,73%)^a,b^	7 ± 8 (10,20%)^a,b^	1 ± 1 (1,27%)^b^
%PF_40_ (measured follicles)	40,00% (80)	42,58% (102)	45,83% (120)	11,67% (120)
CFN	174	87	72	8
**B)**	**Mean number (%)**			
**Follicle group**	**Day 0**	**Day 2**	**Day 7**	**Day 14**
	**(n = 2)**	**(n = 3)**	**(n = 3)**	**(n = 2)**
all follicles	313 ± 123^a^	145 ± 23^a,b^	103 ± 35^a,b^	24 ± 28^b^
shrunken/truncated follicles	29 ± 9 (9,24%)	65 ± 11 (44,55%)	52 ± 20 (49,49%)	8 ± 11 (17,05%)
primordial follicles (%PF)	129 ± 57 (40,82%)^a^	43 ± 8 (29,59%)^a,b^	32 ± 15 (30,35%)^a,b^	10 ± 9 (55,68%)^b^
primary follicles	107 ± 37 (34,32%)	17 ± 5 (11,53%)	11 ± 3 (10,98%)	6 ± 8 (13,64%)
secondary follicles	49 ± 20 (15,62%)^a^	21 ± 3 (14,33%)^a,b^	9 ± 2 (9,08%)^a,b^	1 ± 0 (13,64%)^b^
%PF_40_ (measured follicles)	40,00% (80)	33,33% (120)	24,17% (120)	55,00% (44)
CFN	174	67	43	31

a, b - different subscript indicate for significant differences.

The comparison of two different protein sources revealed that seven days after culture initiation, the loss of %PF_40_ in the BSA supplemented group, with 24% viable PF, was different to 46% PF in FCS cultured pieces (p < 0.001). Although the percentage of viable PF was higher for the BSA group compared to FCS at the end of culture (p < 0.001), the overall amount of follicles found in FCS cultured pieces (120 follicles) was higher compared to the BSA group (44 follicles).

#### Vitamin C supplementation

**Figure 2:**
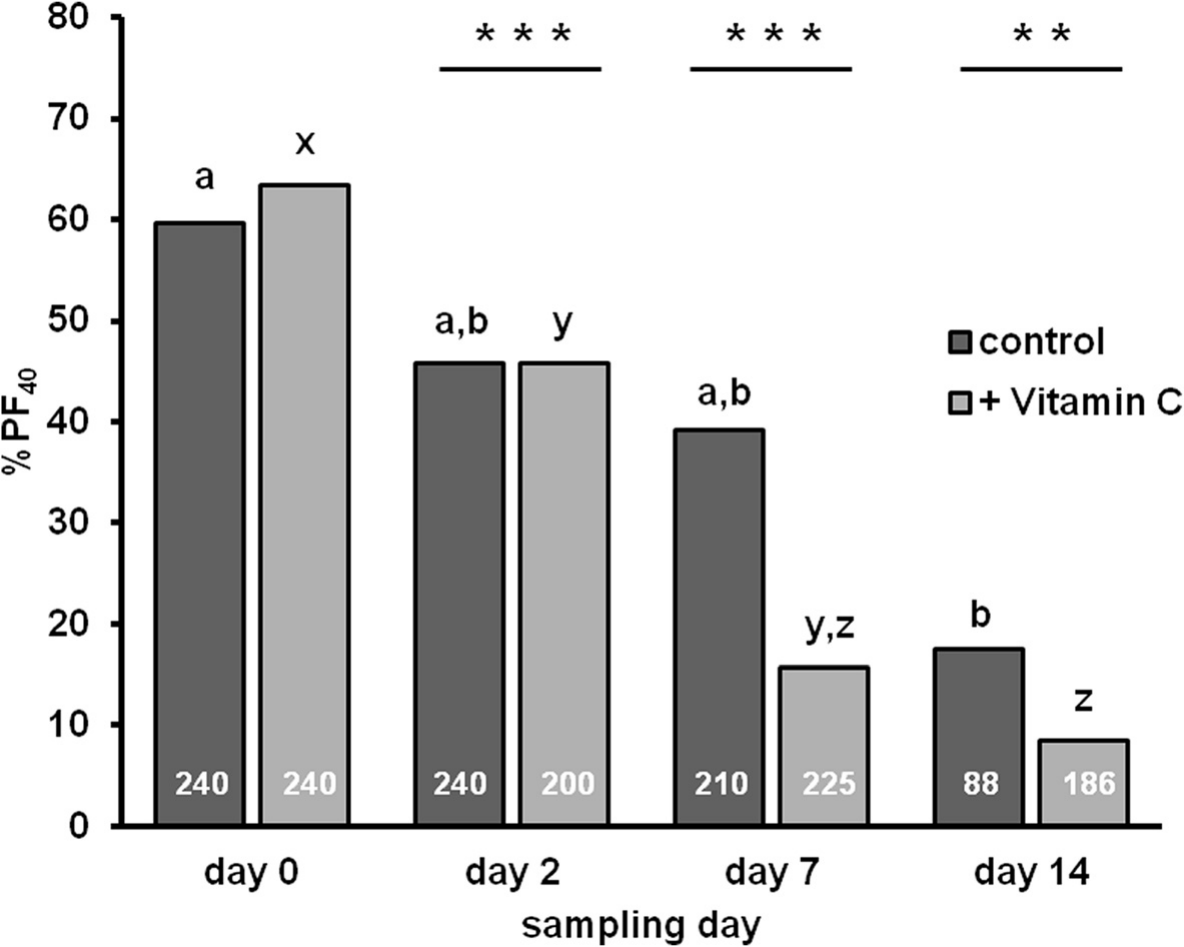
**Percentage of feline primordial follicles after culture with (+ Vitamin C) and without Vitamin C supplementation (control).** The percentage of primordial follicles was determined in 40 follicles (%PF_40_) per ovarian cortex piece from domestic cats. Shown are the mean values at day 0, 2, 7 and 14 of culture. Number of measured follicles are indicated in white letters in the bars. Statistical differences between treatment groups at the same time point are marked with stars p < 0.01 (**), p < 0.001 (***). Different subscript (a, b, c) indicate for significant differences within the control group. Different subscript (x, y, z) indicate for significant differences within the Vitamin C supplementation group.

The effect of Vitamin supplementation was tested in parallel to experiment on the pool of domestic cat ovarian cortex pieces (Table 2). The mean follicle number per piece in the control group did not change during culture, whereas an addition of Vitamin C caused follicular loss from 621 ± 347 at day 0 to 74 ± 37 at day 7 and also to 61 ± 46 at day 14 (p < 0.01, Additional file 1). The shift in the follicular distribution was also evident in the percentage according to all follicles (Additional file 1), and as determined in 40 follicle counts. For clarity, the change in primordial frequency is demonstrated only for 40 follicles per piece (%PF_40_, Figure 2).

Figure 2 demonstrates the impact of Vitamin C on the survival of PF within ovarian cortex pieces obtained from domestic cats during a 14 day culture period. At the beginning (day 0) PF are about 60% of the follicular population. With ongoing time in vitro this amount decreased continuously as demonstrated by the increasing percentage of shrunken follicles (Additional file 1). In the control group, compared to day 0 on day 14 (p < 0.01) a decay was found. In the Vitamin C group, a follicular loss was measureable already on day 7 (p < 0.01, Additional file 1). In addition, the decrease in the survival of PF (%PF_40_) was seen after 2 (p < 0.05), 7 and 14 days of culture compared to the initial follicle number (p < 0.001). Furthermore, the %PF of all follicles found in Vitamin C treated ovarian pieces compared to the second day of culture, and 7 to 14 days, was different (p < 0.05, p < 0.001, Additional file 1). Overall, no Vitamin supplementation (control group) produced higher number of viable PF after 2 days, 7 days and 14 days of culture in comparison to Vitamin C supplementation (p < 0.001, p < 0.01).

### Standard sampling protocol for all other felid species

Table 4 presents the results of ovarian cortex freezing in ten different carnivore species. The CFN indicate that young, small cats (1 to 5 years old: domestic cats, Rusty-spotted cat, Serval) have on average more follicles (367 ± 179) per ovary in comparison to bigger felids of young age (1 to 7 years old: African lion, Amur leopard, Northern Chinese leopard, CFN = 194 ± 89, data not shown in the Table). The animals age also influences the number per piece. The Geoffroy’s cat (99 follicles per piece) and the Oncilla (100 follicles per piece) were quite old at 12 and 13 years, whereas the domestic cats (1 to 4 years, 519 follicles per piece) and 5 year-old Rusty-spotted cat (412 follicles per piece) had a larger oocyte reserve.

**Table 4:** Percentage of viable primordial follicles in ovarian tissue obtained from ten carnivore species

**Species**		**Day 0**		**Day 7**			
				**Fresh**		**Cryo**	
		**%PF**_**40**_	**CFN**	**%PF**_**40**_	**CFN**	**%PF**_**40**_	**CFN**
African lion	(*Panthera leo*)	51% (240)^a^	183	39% (120)^b^	130	51% (80)	208
Amur leopard	(*Panthera pardus orientalis*)	39% (160)	110	46% (120)^a^	72	23% (120)^b^	91
Black-footed cat	(*Felis nigripes*)	-	-	-	-	55% (40)	122
domestic cat	(*Felis silvestris catus*)	57% (360)^a^	519	35% (208)^b^	121	32% (120)	207
Geoffroy’s cat	(*Leopardus geoffroyi*)	31% (120)	99	28% (40)^a^	64	48% (80)^b^	170
Northern Chinese leopard	(*Panthera pardus japonensis*)	49% (120)^a^	288	28% (80)^b^	133	21% (80)	114
Oncilla	(*Leopardus tigrinus*)	43% (200)	100	-	-	44% (80)	93
Rusty-spotted cat	(*Prionailurus rubiginosus*)	54% (200)	412	-	-	41% (80)	182
Serval	(*Leptailurus serval*)	49% (80)	169	48% (40)	137	48% (80)	111
Sumatran tiger	(*Panthera tigris sumatrae*)	41% (200)	193	40% (80)	169	45% (120)	155

a, b - different subscript indicate for significant differences.

%PF_40_ (n measured follicles) and CFN was determined at day 0 and after seven days culture of fresh and frozen-thawed ovarian pieces.

**Figure 3:**
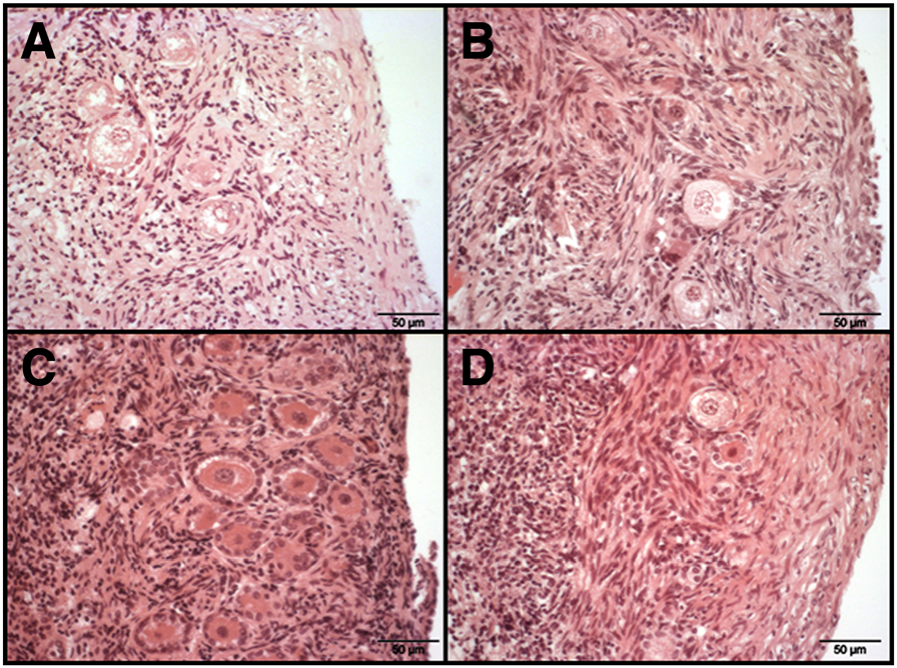
**Histological sections of frozen-thawed ovarian cortex of four non-domestic felids.** Shown are ovarian cortex pieces immediately after thawing at day 0 from **A**) African lion, **B**) Northern Chinese leopard, **C**) Rusty-spotted cat and **D**) Geoffroy's cat. The lower follicle in picture section D is degenerated.

Figure 3 presents the histological sections of four representatives of wild felid species fixed immediately after thawing. The gross morphology did not reveal any differences in follicular structure between the different felid species compared to fresh tissues (pictures not shown) or to the appearance of fresh domestic cat cortex (see Figure 1).

The success of post-thaw survival was assessed by comparison of the CFN and follicular population (%PF_40_) on day 7 of culture before and after cryopreservation. In addition, the impact of culture was determined by comparison the %PF_40_ at day 0 and day 7 of culture. For the domestic cat, the Northern Chinese leopard and the African lion a loss of follicles were found during culture independently of the cryopreservation process (p < 0.001, p < 0.01, p < 0.05). In two (Geoffroy’s cat, Amur leopard), the %PF_40_ after thawing and seven days of culture was different from the fresh cultured follicle values found at day 7. The Geoffroy’s cat tissue samples showed a higher number of viable PF in frozen-thawed samples after 7 days of culture compared to fresh cultured pieces at the same sampling day (p < 0.05). For the Amur leopard, a loss (p < 0.001) of PF was found at day 7 after cryopreservation. There was not enough ovarian tissue available from the other small cats (Black-footed cat, Oncilla, Rusty-spotted cat) to perform the whole set of sampling. Nevertheless, the percentage of PF after freezing and culture indicate a normal population of surviving follicles.

## Discussion

This study used established histological and culture methods to develop a successful cryopreservation protocol for felid species to be used for gamete rescue under Zoo settings.

Very few studies address cryopreservation of the ovarian cortex in cats. Bosch et al. [14] reported the survival of cat ovarian tissue after xenotransplantation into nude mice, whereas Lima et al. [19] and Luvoni et al. [20] assessed the post-thawed survival of cat ovarian tissue by histological evaluation of follicular structure. Our group used BrdU-labeling and Hoechst staining for integrity assessment of mechanical isolated preantral follicles to control culture impacts [21], but it was shown that the procedure of isolation from the ovarian cortex itself can cause a loss of integrity in primordial follicles [22]. We also suggested the application of high-resolution ultrasound to monitor the survival of fresh ovarian xenotransplants in immunodeficient recipients as a prerequisite for oocytes retrieval [15]. The freezing of isolated preantral follicles as well as COCs is not well established yet for felid species. In the present study we focused on preserving ovarian tissue by cryopreservation in conjunction with integrity assessment after culture of frozen-thawed pieces by applying standard histology and a novel aspect of follicle counting (40 follicles per piece) for time saving.

To standardize the histology procedure, similar sized ovarian cortex pieces were obtained using biopsy punches. Within these pieces the follicular number and size distribution was determined during maximal 14 days of in vitro culture. In the domestic cat, the number of preantral follicles per piece was quite variable with mean values of 654 ± 427 (Table 2). Histology of ovarian cortex before culture depicted nicely structured PF (Figure 1, Figure 3) that were not reflective of the dramatic loss of total number that occurred after two days. This change was accompanied by an increased percentage of small (< 30 μm) follicles compared to those of normal size (30 to 40 μm). We suggest that the shrunken follicles were already dead and underwent apoptosis during the culture period. After two days of culture, the overall follicle number dropped down to 419 ± 240, with the percentage of PF reduced by one half. After seven days, about 39% of the overall number were still detectable by histology with 40% intact PF. Therefore, it appears that follicles of different size undergo a similar rate of attrition during culture. The high standard deviation in all follicle groups demonstrates the variation in the number of follicles between ovarian cortex pieces. Culture of ovarian cortex pieces in an agitating system and in addition the use of a large volume of culture medium resulted in healthy follicles after culture, as also described for human tissue [23].

To determine the overall follicle number in each piece, serial sections were performed with at least 240 cuts. But in summary only every tenth cut was used for follicles measurements to avoid a repeat measurement of the same PF. To reduce this work-load for morphological analysis, the percentage of structural intact PF (30 to 40 μm) was determined in a representative number of follicles (40 follicles per piece, 2 to 6 pieces per data point). As shown for the domestic cat pool and the Amur leopard the percentage of PF determined was only 40 per piece and was not different from the percentage of PF from all follicles (Tables 2, 3). If only 40 follicles per piece were measured (all other specimens) the CFN was extrapolated by considering the number of sections to view 30 primordial follicles. As shown in Tables 2, 3 and Figure 2 this methodological simplification (focus count) still reflects the follicle survival during culture and was successfully used to optimize the culture conditions.

In contrast to reports on other species where the growth activation of PF in vitro occurred spontaneously in whole ovary culture of rodents, in cortical tissue culture of cattle [24], baboons [25] and humans [26], we never observed an initiation of follicular growth from PF to more advanced stages. In some species, follicle activation was initiated by supplementation of gonadotropins like FSH and growth factors in caprine [27] and human ovarian cortex [28]. In others, the primary to secondary transition might be increased by Testosterone addition such as in sheep cortical tissue in ovo [29] and in bovine ovarian tissue in vitro [30]. For further investigations we plan to stimulate the follicular growth within the ovarian tissue of feline species by supplementation of gonadotropins or to apply xenotransplantation to circumvent long-term in vitro culture.

In vitro maturation and fertilization in the domestic cat is usually performed by the addition of BSA as the main protein source. Immature cat oocytes do not tolerate the presence of FCS in the culture medium [31] and small preantral follicles prefer BSA [21]. In contrast to isolated feline oocytes, intraovarian PF might depend on the addition of a more complex protein source like FCS during culture. At least we could show for the ovarian cortex pieces of an Amur leopard, the relative PF number was constant during the first seven days of culture in FCS supplemented medium, whereas BSA supplementation resulted in a significant decrease by day 7. The Amur leopard tissue was used for this experiment because we consider it as a representative species for all felids. Experiment 1 and 2 showed that both feline species behave similarly in culture, and differences in survival between all examined species can be mostly related to age, temperature and duration of transportation (Table 4). Due to the large number of ovarian cortex pieces per ovary (~ 100) obtained for the Amur leopard, it is possible to compare different culture conditions with samples collected under the same conditions. In the case of smaller cats (especially for the domestic cat as model organism) only 10 to 16 pieces per ovary are available. For this reason comparative culture or freezing studies can be performed only on a pool of samples, introducing individual variation into the experiment.

Vitamin C was found to be essential for ovarian tissue culture in the goat [32]. It influenced the maintenance of follicular integrity and promoted follicular activation and growth in combination with FSH and/or growth factors. Also in mice [33] and cattle [34] ascorbic acid treatment was involved in follicular growth initiation. For feline immunodeficiency virus-infected cells supplementation of Vitamin C resulted in an inhibition of apoptosis and virus replication [35]. Furthermore, the determination of ill cats showed a high ascorbate concentration in plasma which function as a potential species response to oxidative stress [36]. Our results, however, suggested the detrimental effect of Vitamin C supplementation on cat follicle survival (Figure 2) in the ovarian cortex. Therefore, we omitted this component from our standard post-thawed culture medium.

Based on the results of experiment 1 and 2 we established a standard protocol for post-thawed culture and integrity assessment of intra-ovarian feline follicles which has been applied to ten feline species (Table 4). The culture medium composition was identical to Telfer et al. [17] supplemented with FCS and omitting Vitamin C. No structural differences are seen in histology on day 0 before and after thawing (Figure 3). Therefore, prolonged culture for at least seven days is necessary to reveal any possible impact of cryopreservation on the survival of PF.

The percentage of PF (30 to 40 μm) in tissue samples on day seven of culture in both, fresh and frozen-thawed, ovarian tissue samples was used to assess the cryopreservation outcome. In addition, the initial proportion of PF before culture was determined to correct for the (possible) impact of culture. Only in domestic cat, Northern Chinese leopard and African Lion ovarian samples a significantly decrease of PF between day 0 and 7 in fresh samples was observed. We indicate that loss, as an effect of tissue culture conditions, are still not sufficient to be used for these three feline species.

In all other examined samples, culture had no negative effect on PF survival as shown by identical percentages of PF within the cortex after one week of culture, independent of the cryopreservation procedure. Although we did not have enough material in the three small cats (Black-footed cat, Oncilla and Rusty-spotted cat) to perform the fresh day 7 control, the percentage of viable PF at day 0 fresh and day 7 after freezing was not different. The sole exception was provided by the Geoffroy’s cat and the Amur leopard where we found a significant increase of PF values caused by the huge variation of existent follicles per ovarian cortex piece after seven days post-thaw.

In our study we applied a slow freezing cryopreservation protocol which is successfully used for human ovarian tissue [10]. For feline ovarian cortex, however, only a few reports are available on cryopreservation protocols, both on slow freezing and vitrification. Bosch et al. [14] and Lima et al. [19] used almost identical cooling rates and ethylene glycol as cryoprotective agent as in our study, but they abstained using sugar. In both studies only domestic cat ovarian tissue was used and immediately after thawing 58% and after xenotransplantation only 10% of viable follicles were found. Our data indicated no differences in percentage of follicle numbers before and after cryopreservation. In contrast to Bosch et al. [14] we didn’t perform xenotransplantation of frozen-thawed tissue, but after one week culture still a high amount of follicles were detectable. Other authors vitrified the tissue [20] and reported the survival of oocytes after thawing again without performing culture examination. Therefore, it is very difficult to compare the results and conclude about the best freezing approach for cat species.

Although the CFN presents only a rough estimation of follicles within the ovarian cortex, we are convinced that this parameter reflects the ovarian oocyte reserve of a female animal very effectively. It was shown that the CFN was influenced by age and size of felid species. Even more, the results after thawing and 7 days culture indicate that still a high amount of viable oocytes can be preserved after the death of an animal. If the methods for realization of the oocyte pool can be developed for ART in the future, the frozen ovarian tissue of rare and valuable felid species can serve a resource bank to increase genetical diversity.

## Conclusion

In conclusion, this is the first report of preserving a huge amount of follicles within ovarian tissue by slow freezing performed in several wild felid species. The cryopreservation protocol, which is already successful used for human medicine to restore fertility in cancer patients [9], can be recommended for carnivore species. The post-thawed integrity assessment was critical for the examination of freezing and culture protocols, because serial histological analysis is very time consuming. The suggested focus counting and calculation of follicle number per piece and the primordial proportion from the overall follicular population might be used to compare treatment protocols, although alternative approaches should also be considered. The final proof of follicle survival can be done only by xenotransplantation into immunodeficient recipients [14] and verification of follicular growth.

## Competing interests

The authors do not have any conflicts of interests based on the presenting information and data. In addition, the authors do not have any financial competing interests.

## Authors’ contributions

All authors contributed to the study design. Caterina Wiedemann performed culture, cryopreservation and thawing of ovarian cortex and analyzed the data. CW, JZ and KJ drafted the paper. All authors read and approved the final manuscript.

## Supplementary Material

Additional file 1: Table 2B Follicle distribution in pooled domestic cat ovarian cortex pieces over 14 days in vitro culture with Vitamin C supplementation. Click here for file
